# Simulation of Spread and Control of Lesions in Brain

**DOI:** 10.1155/2012/383546

**Published:** 2012-01-29

**Authors:** Krishna Mohan Thamattoor Raman

**Affiliations:** CSIR Centre for Mathematical Modelling and Computer Simulation (C-MMACS), Bangalore 560017, India

## Abstract

A simulation model for the spread and control of lesions in the brain is constructed using a planar network (graph) representation for the central nervous system (CNS). The model is inspired by the lesion structures observed in the case of multiple sclerosis (MS), a chronic disease of the CNS. The initial lesion site is at the center of a unit square and spreads outwards based on the success rate in damaging edges (axons) of the network. The damaged edges send out alarm signals which, at appropriate intensity levels, generate programmed cell death. Depending on the extent and timing of the programmed cell death, the lesion may get controlled or aggravated akin to the control of wild fires by burning of peripheral vegetation. The parameter phase space of the model shows smooth transition from uncontrolled situation to controlled situation. The simulations show that the model is capable of generating a wide variety of lesion growth and arrest scenarios.

## 1. Introduction

MS affects about one million people worldwide and causes physical and cognitive disability. There are three types of MS, relapsing-remitting, secondary progressive, and primary progressive, that differ in the dynamical patterns of disease progression. There are as yet no known cures for MS. Patients with relapsing MS are currently treated with drugs that exert immunomodulatory effects and slow the progression of the disease; there are no effective treatment options for the progressive forms of MS [1, 2].

MS is postulated to be a cell-mediated autoimmune disease directed against myelin components of the CNS. Myelin is an electrically insulating phospholipid layer that surrounds the axons of many neurons. The disease is characterized by both inflammatory immune responses and neurodegeneration. The prevailing hypothesis on MS pathogenesis is that autoreactive T-lymphocytes, a cell type in the immune system, orchestrate a complex cascade of events that cause blood-brain barrier disruption and invasion of immunologically aggressive cells into the CNS. However, the exact causes of MS still remain unknown [3, 4]. The long-term goals of this research are to develop disease models that can be used to evaluate therapeutic strategies for this disease and, in this report, the specific focus is on evaluating a network model for MS lesion dynamics. Literature survey indicates that network approaches have not been studied extensively for disease modeling in MS.

### 1.1. Previous Work

Conventional models for autoimmunity are premised on the occurrence of defects in the immune system that cause it to turn against the host tissue. A defect-free immune system, in this world view, purportedly only attacks pathogens, the external agents that cause illness or disease [5–7]. However, an alternative viewpoint has been advocated where auto-immunity is seen as the usual immune response, but directed against those components of the body which, in normal conditions, are inaccessible to the immune system [8–14]. For example, in the *danger model*, developed by Matzinger [10, 11], it is posited that stressed and injured tissues can mediate immune responses through the generation of appropriate “danger” signals. This is as opposed to the activation through recognition of external pathogenic cell types from host tissue in the conventional models. The concept of *comprehensive immunity*, developed by Nevo et al. [12, 13] complements this alternate perspective; experimental results supporting their idea have also been reported [14]. The present network model is inspired by the alternative viewpoint.

The key elements of the model consist of a pathological process that causes cellular damage and programmed cell death (apoptosis) initiated through an intercellular signaling component. The programmed cell death deprives the pathological process of healthy tissue which is necessary for its propagation in space and time. In this, it resembles the action of firemen who burn peripheral vegetation to contain forest fires. Inter-cellular signaling is a key feature of the model that allow pathologically damaged cells to propagate alarm signals and initiate programmed cell death.

## 2. Model

An undirected, fixed radius random graph *G*(*n*, *r*), with *n* nodes (vertices) and radius of connectivity, *r*, is constructed to represent the CNS in this 2D network model. Fixed radius implies that nodes are connected only if they are within a distance of *r*. Biologically, the nodes of the graph can be viewed as representing cell bodies or functional units and the edges (bonds) of the graph can be viewed as axons or the interconnections between functional units.

Let *d*_*i*_ be the degree of the *i*th node, that is, the number of edges attached to it. The health status of each edge, at time *t*, is indicated by its “weight,” *w*(*j*, *t*)(*j* = 1,…, *d*_*i*_), an integer number ranging from 0 ⋯ *w*_max⁡_. Edges with weight *w*_max⁡_ are fully functional or healthy units (as at the beginning of simulation), and those with weight zero, are dead. Extending the same logic, the amplitude of the signal propagated along *j*th edge is taken to be equal to *w*(*j*, *t*).

In the pathological process, the edges are damaged by lowering their weight by a single unit. However, in the programmed cell death process, edge weights are directly reduced to zero. In the regeneration process, edge weights are raised by a unit.

The pathological and regeneration processes are driven by probabilistic events wherein each edge in the affected region, in each time unit, has a certain probability **p**_*i*_^*d*^(**p**_*i*_^*r*^) of getting damaged (regenerated). In the general case, **p**_*i*_^*d*^(**p**_*i*_^*r*^) is a column vector of length *w*_max⁡_ containing the transition probabilities from one state of health to another. Probability of programmed cell death, *p*_*p*_, is independent of the health status of the edge.

The functional or health status of the *i*th node is the sum over its edge weights, *s*_*i*_(*t*) = ∑_*j*=1_^*d*_*i*_^ *w*(*j*, *t*). The maximum possible value of *s*_*i*_ is denoted by *S*_*i*_, which is realized when each *w*(*j*, *t*) = *w*_max⁡_.

A node damaged by the pathological process generates an alarm signal when the ratio of its health status to the fully healthy state (*s*_*i*_(*t*)/*S*_*i*_) falls below a threshold, *τ*_*al*_. The signals received at the *i*th node are summed and propagated further when the summed signal strength reaches *s*_*i*_.

Programmed cell death is initiated at all the nodes where the propagated signals reach a threshold *τ*_*bf*_. The accumulated alarm signals in the region of programmed cell death, a circular region around the activated node of radius proportional to a parameter *C*_*bf*_, get reset to zero. No additional signals are generated at these nodes to the alarm signals generated in the pathologic process.

The spread of the pathologic process is driven by the success rate in causing cellular damage. The fraction of edges (*R*_*I*_(*t*)) damaged in a particular time step, among the total number of healthy edges visited, is the rate of damage due to the pathologic process. The rates of damage due to the pathologic and the programmed cell death are computed in terms of the initial lesion size so that the final results are invariant with respect to the initial lesion size. Thus, the radius of the region affected by the pathologic process increased or decreased according to the formula, *α* × *R*_*I*_(*t*) × *ROI*_*t*=0_, where *ROI*_*t*=0_ is the radius of the region at the center where the initial lesion is seeded. In a similar fashion, the region of programmed cell death was computed as *C*_*bf*_ × *ROI*_*t*=0_.

## 3. Simulation

In the simulations reported here, a two-state model with *w*_max⁡_ = 1 has been employed, that is, there are no intermediate states of health, and the edges are either alive or dead. Additionally, the regeneration probability, **p**_*i*_^*r*^, was set to zero in order to focus exclusively on the effects of the interplay between the pathological and programmed cell death processes on lesion structure and dynamics. A few preliminary results using such a configuration was reported earlier [15].

We have set *n* = 400 and chosen a uniform random distribution of points in the unit square [0,1]×[0,1]. The radius of connectivity was set to *r* = 0.2. All the results were also confirmed on a network of *n* = 4000, with *r* = 0.06. Average degree strengths of the order of 10 are obtained in these configurations; degree distribution is Gaussian. The pathological process was initiated at *t* = 0 in a region with *ROI*_*t*=0_ = 0.05 around the center at (0.5, 0.5); for *n* = 4000, *ROI*_*t*=0_ = 0.015.

A uniform probability of pathologic damage *p*_*d*_ = 0.33 was used, with *α* = 0.12. We varied *τ*_*al*_, *τ*_*bf*_, and *C*_*bf*_ to identify the conditions under which the pathological process could be controlled by the programmed cell death. Larger values of *τ*_*bf*_ indicate reduced sensitivity to the alarm signals whereas a larger value of *C*_*bf*_ indicates that a larger area near the alerted node is subjected to programmed cell death. In the case of *τ*_*al*_, larger values indicate quicker firing of alarm signals.

## 4. Results

**Figure 1:**
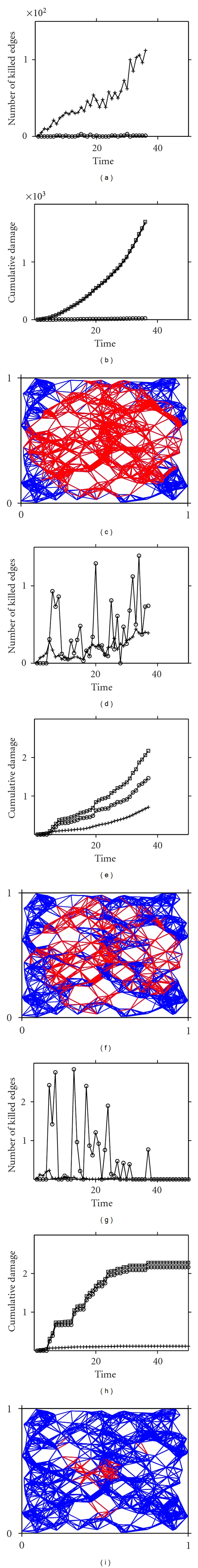
Time course of damages to the system by the pathological and programmed cell death processes. The first column of panels shows, separately, the instantaneous damages due to both the processes; dotted lines with asterisks indicate the damages due to the pathological process, and bold lines with filled circles indicate damages due to programmed cell death. The second column of panels shows the cumulative damages with time. Again, the damages effected through both the processes have been separately shown (same symbols as earlier), as also the sum total damages to the system (square symbol). The last column of panels (color online) show the state of the network at the end of the simulations; the dotted lines indicate healthy edges (axons), full (blue) lines indicate edges damaged due to programmed cell death, and dark (red) lines indicate edges damaged due to the pathological process. For all the panels, *τ*_*bf*_ = 0.5 and *τ*_*al*_ = 0.7, while the *C*_*bf*_ values, for each row, top to bottom, are 0.2, 0.8, and 1.5, respectively.

Figure 1 shows the time series of damages caused to the system by both the pathological process and the programmed cell death process. The first column of panels in the figure shows the time course of instantaneous damages to the system. The middle column of panels shows the time course of the cumulative damages to the system. The last column of panels show the final state of the network at the end of the simulations.

There are three typical scenarios which are illustrated in Figure 1, in the three rows from top to bottom. Figures 1(a)–1(c) show a scenario where the programmed cell death is not of sufficient strength to significantly affect the pathological process. Note that the instantaneous damages from programmed cell death are hardly ever above zero. Also, it is seen from Figure 1(b) that the contribution of programmed cell death to the sum total of damages is insignificant. This situation occurs with a suitable combination of low *τ*_a*l*_, high *τ*_*bf*_, and low *C*_*bf*_ values. Figures 1(d)–1(f) show a slightly more complex situation. In this case, programmed cell death is clearly the dominant effect. The instantaneous damages caused by both the processes are consistently nonzero (Figure 1(d)) and the cumulative damages (Figure 1(e)) continue to grow. The total damage, thus, continues to spread. In Figures 1(g)–1(i), the pathological process has been well controlled. The instantaneous damages have fallen to zero in Figure 1(g), and the cumulative damages (Figure 1(h)) have leveled off. The final state of the network (Figure 1(i)) shows that the damage is also minimal in terms of the fraction of edges damaged.

**Figure 2:**
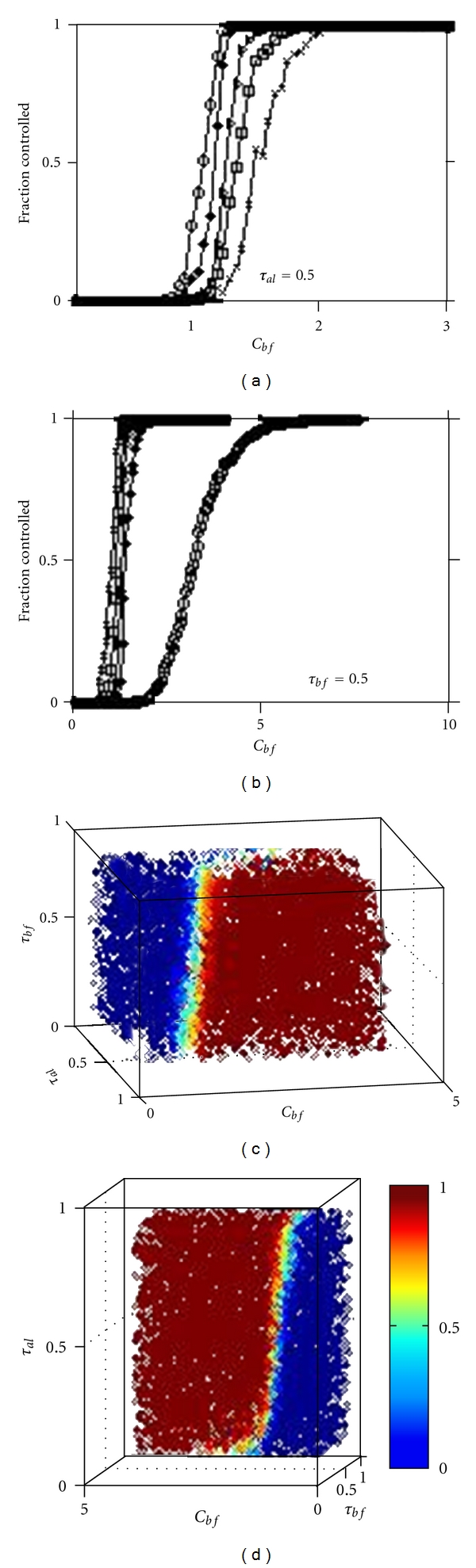
Phase portraits of the dynamics. The *y*-axes in Figures 2(a)-2(b) and the gray (color online) scale in Figures 2(c)-2(d) indicate the fraction of the total number of simulations where the pathological process was successfully arrested. In Figure 2a, *τ*_*al*_ has been held fixed at the shown value while the different curves are, from left to right, for *τ*_*bf*_ = 0.1, 0.3, 0.5, 0.7, and 0.9. In Figure 2(b), the different curves, from right to left, are for *τ*_*al*_ = 0.9, 0.7, 0.5, 0.3, and 0.1; *τ*_*bf*_ = 0.5 is held fixed. The *S*-curves that result with increasing *C*_*bf*_ values indicate the smooth nature of the transition in dynamical behavior in the parameter space.

As seen from Figures 1(a), 1(d), and 1(g), the time series is stochastic. There are essentially two sources of randomness in the model. Firstly, the pathological process is simulated by a binomial process wherein each edge visit will lead to successful damage if the generated random number falls below the value in **p**_*i*_^*d*^ for that edge. Secondly, the random network itself is generated by the random distribution of the *n* points in the plane. The complete picture of the transition from uncontrolled growth of the pathological process to the situation where the pathological process has been well arrested is seen in the parameter phase space graphs shown in Figure 2, where an averaging has been effected over the two sources of randomness. The phase space diagrams are the results of averaging over ten different networks, with the dynamics averaged over a thousand iterations.

From Figure 2, we see that the transition from uncontrolled pathological process to arrested pathological process is smooth as *C*_*bf*_ is varied from low to high values. In Figure 2(a), *τ*_*al*_ has been held fixed and the different curves, from left to right, are for different *τ*_*bf*_ values, from 0.1 to 0.9 in steps of 0.2. In Figure 2(b), *τ*_*bf*_ has been held fixed and the different curves are, from right to left, for *τ*_*al*_ values ranging from 0.1 to 0.9, in steps of 0.2. We shall denote by *C*_*bf*_^*cr*^(*τ*_*al*_, *τ*_*bf*_) the critical value of *C*_*bf*_ at which these *S*-curves attain a value of 1, that is, all instances of simulations result in the growth of pathological process being arrested. The combined picture in the three parameter space is presented in Figures 2(c)-2(d), from two different perspectives. The three different scenarios presented, from top to bottom, in Figure 1 indicate, respectively, the three different parts, from left to right, of a typical *S*-curve of Figures 2(a)-2(b).

**Figure 3:**
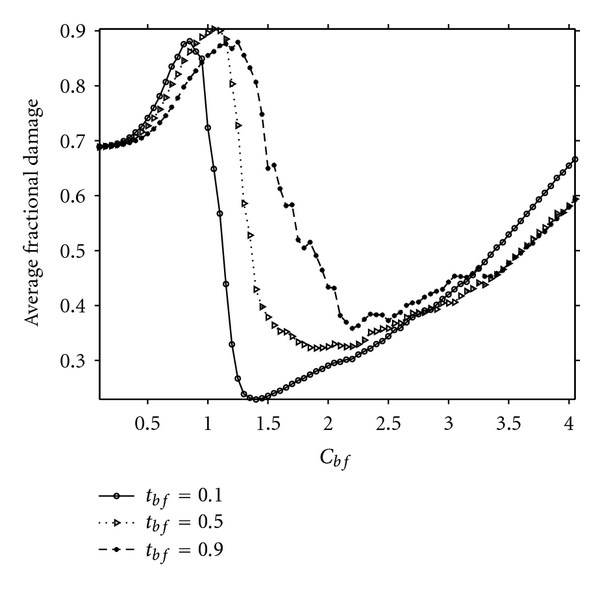
Optimality in sum total damage to the system while effecting arrest of pathological process is shown here. With increasing *C*_*bf*_ values the minimum in sum total damage to the system occurs at about the same value as the critical value at which the fraction of simulations in which the pathological process is arrested attains unity (cf. Figure 2(a)). This minimum occurs at higher values of *C*_*bf*_ with larger *τ*_*bf*_, and the minimum value also shifts upward. A similar situation occurs with different *τ*_*al*_ values (not shown here). Note that the *y*-axis values have been taken at *t* = 20.

As seen from Figure 2, pathological process is always controlled if *C*_*bf*_ > *C*_*bf*_^*cr*^. Nevertheless, the sum total damage to the system is not the same for all values of *C*_*bf*_ > *C*_*bf*_^*cr*^; in fact, the damage is greater, the larger the value of *C*_*bf*_. Clearly, it is desirable to effect control of the pathological process with the least sum total damage to the system. With this in mind, average fractional damages at different *C*_*bf*_ values have been plotted in Figure 3. Three different curves for three different *τ*_*bf*_ values are shown in this figure; similar graphs can be constructed for different *τ*_*al*_ values as well (not shown here; see [16]). These averages have been taken at *t* = 20 in each case. For *C*_*bf*_ < *C*_*bf*_^*cr*^ values, the damages due to the pathological process as well as sum total damage are still growing and have not become stationary at *t* = 20; for *C*_*bf*_ > *C*_*bf*_^*cr*^ values, the averages have become stationary. Nevertheless, these curves indicate that the least sum total damage to the system, with pathological process arrested, is obtained at *C*_*bf*_ = *C*_*bf*_^*cr*^. For *C*_*bf*_ > *C*_*bf*_^*cr*^, the programmed cell death is clearly effecting more damage than is necessary to arrest the pathological process. Since *C*_*bf*_^*cr*^ depends on *τ*_*bf*_ and *τ*_*al*_, it is not surprising to see that (cf. Figures 2(a) and (3)) lesser damage results when *τ*_*bf*_ is small.

From the above, it is clear (cf. Figures 1(d)-1(e)) that arrest of the pathological process does not necessarily occur if the damage due to the programmed cell death process is greater than the pathological process. What is necessary [16] is that the programmed cell death process be able to encircle the region affected by the pathological process, and, furthermore, be able to create an envelope region of sufficient thickness to offset its likely growth factor, *α* × *R*_*I*_(*t*) × *ROI*_*t*=0_. This is achieved in all instances of simulation when *C*_*bf*_ > *C*_*bf*_^*cr*^. Currently, mathematical analysis of this feature is being carried out to establish the relationship of *C*_*bf*_^*cr*^ with *τ*_*al*_ and *τ*_*bf*_, and the results will be reported soon.

## 5. Conclusions

A physically motivated 2D network model was developed for the CNS and employed to study the process of lesion formation and spread in MS. Intercellular signalling of distress by the damaged cells is a key feature of the model which leads to programmed cell death getting activated in an attempt to arrest the lesion progress. The model demonstrates that the spread of the pathologic process can be arrested by programmed cell death when the geometry of the damage inflicted by the latter leads to an envelope, of sufficient thickness, being created encircling the area of pathological process. Such an envelope of dead cells deprives the pathological process of healthy cells which can sustain its growth. The model shows a smooth transition, as parameters are varied, from the situations of run-away pathological process, through aggravated damage to the system caused by unsuccessful firing of programmed cell death, to the creation of successful envelope around the pathological process.

The model complements the alternate viewpoint on autoimmunity which posits that cells and tissues signal distress and activate the immune system. Such a viewpoint circumvents the need for the immune system to store information about likely pathogens and, also, makes it capable of acting in instances of cellular damage resulting from nonpathogenic causes. Further study of the model along with identification of the possible biological constituents should enable comparisons with experiments and a more detailed exposition.

## References

[1] Kantarci O, Wingerchuk D (2006). Epidemiology and natural history of multiple sclerosis: new insights. *Current Opinion in Neurology*.

[2] Murray TJ (2006). Diagnosis and treatment of multiple sclerosis. *British Medical Journal*.

[3] Sospedra M, Martin R (2005). Immunology of multiple sclerosis. *Annual Review of Immunology*.

[4] Perry VH, Anthony DC (1999). Axon damage and repair in multiple sclerosis. *Philosophical Transactions of the Royal Society B: Biological Sciences*.

[5] Burnet FM, Fenner F (1949). *The Production of Antibodies*.

[6] Burnet FM (1976). *Immunology: Readings from Scientific American*.

[7] Christ E, Tauber AI (1999). Selfhood, immunity, and the biological imagination: the thought of Frank Macfarlane Burnet. *Biology and Philosophy*.

[8] Nossal GJV (1978). *Antibodies and Immunity*.

[9] Smirnova OA, Stepanova NV (1975). Mathematical model of autoimmunity. *Biofizika*.

[10] Matzinger P (1994). Tolerance, danger, and the extended family. *Annual Review of Immunology*.

[11] Matzinger P (1998). An innate sense of danger. *Seminars in Immunology*.

[12] Nevo U, Kipnis J, Golding I (2003). Autoimmunity as a special case of immunity: removing threats from within. *Trends in Molecular Medicine*.

[13] Nevo U, Golding I, Neumann AU, Schwartz M, Akselrod S (2004). Autoimmunity as an immune defense against degenerative processes: a primary mathematical model illustrating the bright side of autoimmunity. *Journal of Theoretical Biology*.

[14] Moalem G, Leibowitz-Amit R, Yoles E, Mor F, Cohen IR, Schwartz M (1999). Autoimmune T cells protect neurons from secondary degeneration after central nervous system axotomy. *Nature Medicine*.

[15] Krishna Mohan TR, Sen S, Ramanathan M (2008). A computational model for lesion dynamics in multiple sclerosis of the brain. *International Journal of Modern Physics E*.

[16] Krishna Mohan TR Unpublished results.

